# Chinese Clinical Named Entity Recognition With Segmentation Synonym Sentence Synthesis Mechanism: Algorithm Development and Validation

**DOI:** 10.2196/60334

**Published:** 2024-11-21

**Authors:** Jian Tang, Zikun Huang, Hongzhen Xu, Hao Zhang, Hailing Huang, Minqiong Tang, Pengsheng Luo, Dong Qin

**Affiliations:** 1 Department of Pharmacy, People's Hospital of Guilin, 12 Wenming Road, Guilin, 541000, China, 86 18978320258; 2 School of Science and Technology, Guilin University, Guilin, China

**Keywords:** clinical named entity recognition, word embedding, Chinese electronic medical records, RoBERTa, entity recognition, segmentation, natural language processing, AI, artificial intelligence, dataset, dataset augmentation, algorithm, entity, EMR

## Abstract

**Background:**

Clinical named entity recognition (CNER) is a fundamental task in natural language processing used to extract named entities from electronic medical record texts. In recent years, with the continuous development of machine learning, deep learning models have replaced traditional machine learning and template-based methods, becoming widely applied in the CNER field. However, due to the complexity of clinical texts, the diversity and large quantity of named entity types, and the unclear boundaries between different entities, existing advanced methods rely to some extent on annotated databases and the scale of embedded dictionaries.

**Objective:**

This study aims to address the issues of data scarcity and labeling difficulties in CNER tasks by proposing a dataset augmentation algorithm based on proximity word calculation.

**Methods:**

We propose a Segmentation Synonym Sentence Synthesis (SSSS) algorithm based on neighboring vocabulary, which leverages existing public knowledge without the need for manual expansion of specialized domain dictionaries. Through lexical segmentation, the algorithm replaces new synonymous vocabulary by recombining from vast natural language data, achieving nearby expansion expressions of the dataset. We applied the SSSS algorithm to the Robustly Optimized Bidirectional Encoder Representations from Transformers Pretraining Approach (RoBERTa) + conditional random field (CRF) and RoBERTa + Bidirectional Long Short-Term Memory (BiLSTM) + CRF models and evaluated our models (SSSS + RoBERTa + CRF; SSSS + RoBERTa + BiLSTM + CRF) on the China Conference on Knowledge Graph and Semantic Computing (CCKS) 2017 and 2019 datasets.

**Results:**

Our experiments demonstrated that the models SSSS + RoBERTa + CRF and SSSS + RoBERTa + BiLSTM + CRF achieved *F*_1_-scores of 91.30% and 91.35% on the CCKS-2017 dataset, respectively. They also achieved *F*_1_-scores of 83.21% and 83.01% on the CCKS-2019 dataset, respectively.

**Conclusions:**

The experimental results indicated that our proposed method successfully expanded the dataset and remarkably improved the performance of the model, effectively addressing the challenges of data acquisition, annotation difficulties, and insufficient model generalization performance.

## Introduction

Named entity recognition (NER) is an important subtask in natural language processing [1]. Its primary function is to identify and classify entities such as diseases in textual data. In the clinical domain, clinical NER (CNER) is used to recognize and classify clinical textual data such as diseases, symptoms, treatments, tests, body parts, and medications in electronic medical records (EMRs) [2]. CNER is mission critical for building intelligent medical assistive systems, such as clinical decision support systems, and constructing medical knowledge graphs [3]. However, clinical text data are usually unstructured, and clinical text syntax might be incomplete with poor contextualization. Clinical terms may have different meanings in different contexts, and this variability and ambiguity make the identification and classification of named entities extremely challenging, thus making NER in the clinical domain more challenging compared to NER in the general domain [4]. Additionally, Chinese EMRs will appear to be more complicated compared to those written in Roman alphabet languages due to the complexity of Chinese grammatical structure and clausal rules [5]. With a relatively flexible word order, the subject-verb-object sequence of the Chinese language depends on the emphasis of the content. In contrast, the sentence structure in Roman alphabet languages is relatively fixed, where the word order has minimal impact on semantics. In Chinese, subjects, objects, or other components are frequently omitted, which poses additional challenges for tasks like NER, as this requires interpreting and adding this missing information. In Roman alphabet languages, sentence components are typically expressed explicitly and omissions are less common. Even when omissions do occur, verb conjugations generally provide sufficient contextual clues. In Chinese EMRs, technical terminology and colloquial descriptions are often interwoven, and the frequent use of polysemy and vague expressions further contributes to linguistic diversity and complexity.

Over the past decade, remarkable advancements have been made in the field of CNER [6-8]. Although conventional dictionary-based techniques can identify names and distinct clinical concepts with high accuracy and precision in matching, the quality and size of dictionaries directly impact recognition outcomes. With the development of machine learning, the theoretical basis for several unsupervised learning algorithms revolves around the distributional hypothesis proposed by Zellig Harris [9]. This hypothesis posits that words with similar semantic meanings tend to appear in coherent contexts. Consequently, these algorithms assign vector representations to words based on their contextual associations. Two notable examples of such algorithms that use the distributional hypothesis are GloVe and word2vec. Word2vec relies on prediction models, while GloVe is based on count-based calculations.

CNER presents increased complexity and challenges. This is due to the widespread use of unconventional abbreviations and various representations of the same entities within the Chinese language. These factors greatly impede the accurate and efficient extraction of crucial information. To address this challenge, dictionary-based approaches require a deep understanding and thorough utilization of well-annotated data sources and relevant knowledge bases. This approach enhances model performance and generalizability.

The adoption of deep learning has led to the emergence of numerous models using a variety of approaches. One such example is the work conducted by Li et al [10], who utilized a lattice long short-term memory (LSTM) model incorporating contextualized character representation for recognizing clinical named entities in Chinese. They developed a novel variant of contextualized character representation and incorporated a conditional random field (CRF) layer into their model. Xu et al [11] introduced a novel neural network approach referred to as dictionary-attention-Bidirectional LSTM-CRF (Dic-Att-BiLSTM-CRF) for disease NER. Their method involved applying an efficient and precise string-matching technique to identify disease entities with disease dictionaries constructed from the disease ontology. Furthermore, Dic-Att-BiLSTM-CRF created a dictionary attention layer by integrating disease dictionary matching strategies and document-level attention mechanisms. Wang et al [12] constructed a dictionary- and context-based approach using medical literature to construct feature vectors for each Chinese character in their proposed combination method of knowledge-driven dictionary methods and data-driven deep learning for NER tasks. The results showed that this approach effectively improved the processing of rare entities; as the size of the dictionary increased, the performance of the method gradually improved.

Despite significant advancements in these methods, several limitations remain. The performance of these approaches relies to some extent on the annotation and embedding capabilities of the underlying databases [13]. Medical datasets often encounter challenges in data collection and annotation, and concerns regarding patient privacy protection and compliance contribute to smaller document collections. Moreover, rarer diseases, drugs, and entities occur less frequently, making it difficult to train models effectively. Few existing methods are universally applicable across diverse datasets, and the generalization performance of the models requires further enhancement due to the peculiarity of medical texts. EMRs abound with ambiguous terms, nonstandard abbreviations, and variations of the same entity, for example, “奥沙利铂(oxaliplatin)” and “奥沙利柏(oxaliplatin)” [14] and “心肌梗死(Myocardial Infarction)”and “心肌梗塞(Myocardial Infarction).” Doctors’ writing styles differ significantly, leading to intricate text structures and challenging comprehension. Current NER tasks in the medical domain are primarily focused on Chinese NER, which presents a challenge due to unclear entity boundaries and difficulties in Chinese word segmentation, thereby undermining model performance.

Based on the above problems, this paper proposes a Segmentation Synonym Sentence Synthesis (SSSS) algorithm based on proximity lexical expressions, which was extensively validated on the China Conference on Knowledge Graph and Semantic Computing (CCKS) 2017 and 2019 datasets. The main contributions of this paper are as follows:

1. We propose an adaptive SSSS algorithm for dataset optimization, which exploits existing public knowledge without manually expanding specialized domain dictionaries. It achieved proximity expansion expression of the dataset through lexical cuts, recombined by substituting new proximity repertoires from vast natural language data.
2. By expanding the proximity vocabulary, our algorithm successfully extended the documents of CCKS-2017 and CCKS-2019 by approximately 17 and 20 times, respectively.
3. We evaluated the algorithm’s performance on CCKS-2017 and CCKS-2019 and achieved relatively competitive results compared to other state-of-the-art models. By extending the proximity vocabulary, our models (SSSS + Robustly Optimized Bidirectional Encoder Representations from Transformers Pretraining Approach [RoBERTa] + CRF and SSSS + RoBERTa + Bidirectional Long Short-Term Memory Network [BiLSTM]+ CRF) outperformed both Bidirectional Encoder Representations from Transformers [BERT] + CRF and BERT + BiLSTM + CRF models in handling unknown and low-frequency entities.

## Methods

### Generating an Extended Dataset Based on Proximal Vocabulary

In our experiment, it was observed that specific entities related to “disease” and “therapy” were relatively scarce compared to other categories in the training dataset. This imbalance in entity distribution may weaken the model’s effectiveness when dealing with rare or subtle mentions of these topics in the medical field. Additionally, given the complexity and uniqueness of the medical domain, creating comprehensive dictionaries requires substantial engineering efforts and expertise from professionals to ensure smooth execution.

In this work, we drew inspiration from the concept of proximal lexical expressions [15] and proposed a method called SSSS. The implementation of this algorithm involved several steps. First, text segmentation was performed using the Jieba library. Then, based on the natural language word library trained with Word2Vec, synonyms were searched and processed using the Synonyms database. Finally, these identified synonyms were integrated into the original training set at appropriate positions.

**Figure 1.**
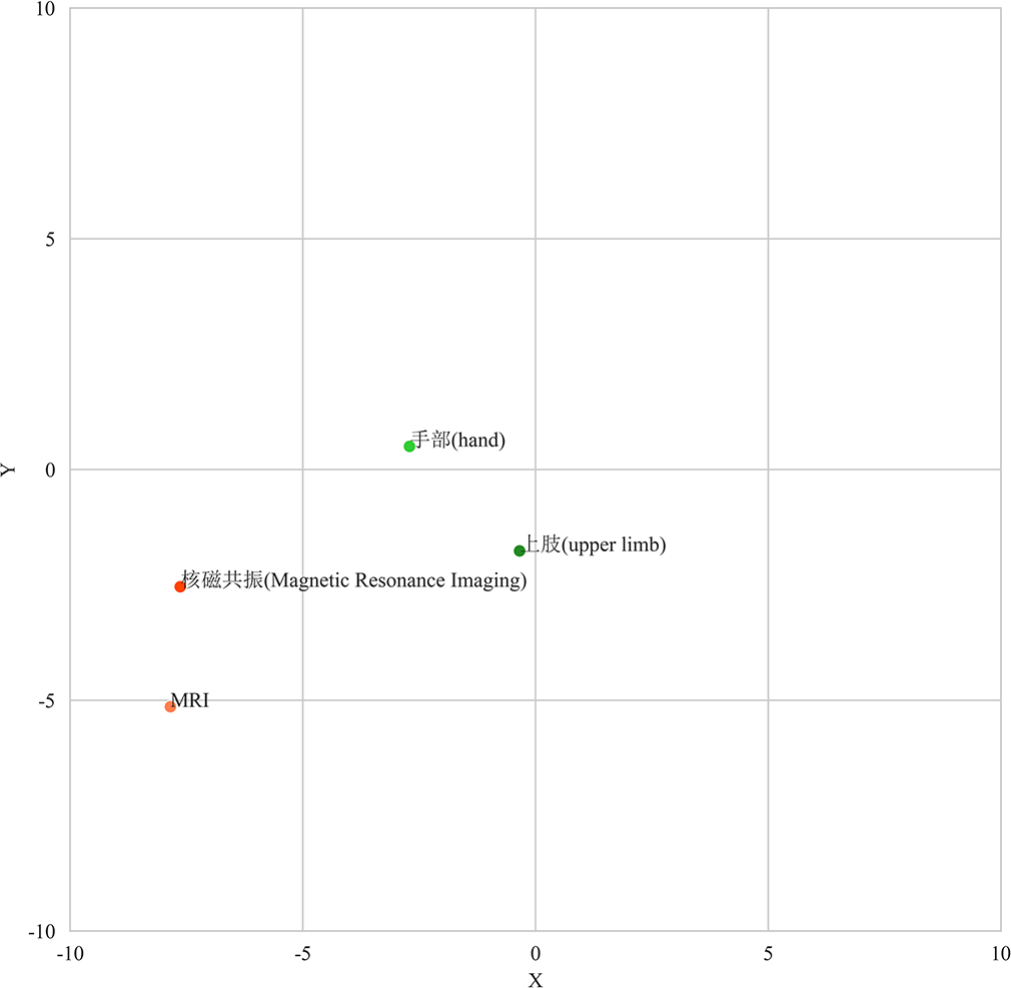
Two-dimensional spatial representation of sample vocabulary.

Specifically, when entity X appeared in the training data, we first used the Jieba library to divide it into multiple simple words, such as X1, X2, and X3. If the number of simple words for an entity exceeded 2, we used the edit distance algorithm to search for synonyms related to it in the Synonyms database [16]. For example, “Norfloxacin” can be associated with its synonym “Fluoroquinolones,” which are different names for the same drug. Additionally, we replaced the original simple words in the processed sentences with the identified synonyms and then reassembled these new complex words to generate synthetic sentences. For instance, after breaking down “Pelvic MRI” into “Pelvis” and “MRI,” we reconstructed them into a sentence using their corresponding synonyms: “Pelvic nuclear magnetic resonance examination.” Through these steps, our aim was to enhance the diversity and richness of the training data, which may contribute to improving the final model’s generalization ability and accuracy. The replaced vocabulary was reintegrated into the surrounding context sentences, aiming to supplement more sentence expressions and vocabulary information without altering the original meaning of the sentences. In similarity calculations, only segmented words were considered; after dimensionality reduction using principal component analysis, they were visualized in a 2D space as shown in Figure 1.

To improve the generalizability and adaptability of models faced with restricted training datasets, this algorithm explored various synonymous or interchangeable wordings while retaining the primary connotations of words. This strategy enabled the expansion of the training dataset size without the need for additional domain-specific dictionaries, thereby reducing reliance on input from domain experts. Consequently, both the workload of domain expertise personnel and the labeling workforce required for datasets were significantly reduced. By implementing this approach, we utilized the SSSS algorithm to enhance the information and vocabulary within the training set, thereby improving the model’s learning ability. Table 1 presents some examples.

**Table 1.** Examples of Segmentation Synonym Sentence Synthesis algorithm expansion.

Entity types	Sentence	Entity	Postexpansion entity
Body	右手中指疼痛不适 (Pain and discomfort in the right middle finger)	右手中指 (Right middle finger)	右中指 (Right middle finger)
Symptom	主因头部外伤出血伴头昏3.5小时入院 (The patient was admitted due to head trauma with bleeding and dizziness for 3.5 hours)	头昏 (Dizziness)	头晕 (Dizziness)
Exam	心电图, 颈动脉彩超等检查 (Electrocardiogram, carotid artery Doppler ultrasound, and other tests)	心电图 (Electrocardiogram), 颈动脉彩超 (Carotid artery Doppler ultrasound)	心电图 (Electrocardiogram), 双侧颈动脉彩超 (Bilateral carotid artery Doppler ultrasound)
Treatment	给予静点头孢哌酮, 炎琥宁联合抗感染 (Administered intravenous cefoperazone and ibuprofen for combined anti-infection treatment)	头孢哌酮 (Cefoperazone), 炎琥宁 (Ibuprofen)	头孢哌酮舒巴坦钠 (Cefoperazone and sulbactam sodium), 炎琥宁 (Ibuprofen)

### Models

#### BERT and RoBERTa

**Figure 2.**
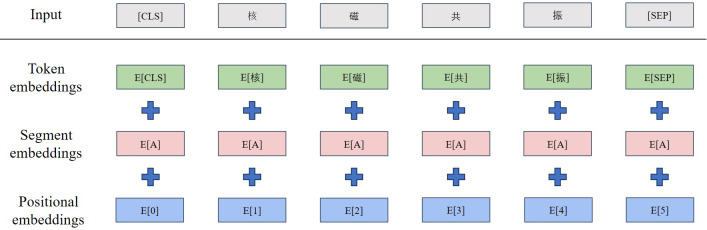
BERT and RoBERTa model structure diagram. BERT: Bidirectional Encoder Representations from Transformers; RoBERTa: Robustly Optimized Bidirectional Encoder Representations from Transformers Pretraining Approach.

BERT [17] is an outstanding pretrained model for text vector representation. Comprising multiple layers of bidirectional transformer encoders, it has the capability to consider the words both before and after a given word, enabling it to ascertain the word’s meaning within the context. The structure of the BERT model is illustrated in Figure 2. This model is obtained through unsupervised task training on a vast corpus of everyday language. It leverages the self-attention mechanism embedded in its encoder layers to learn enhanced word feature representations, which can be directly applied to downstream tasks. However, due to the less frequent occurrence of medical terms in everyday language corpora and the inclusion of more long-tail vocabulary, such as specialized terminologies, it is essential to conduct secondary training on supervised medical corpora for downstream tasks. RoBERTa [18], developed by Facebook, is a derivative version of the original BERT model. It inherits BERT’s basic architecture, including stacked transformer layers and bidirectional context encoding. It enhances the training set’s variability through dynamic masking in language modeling, improving the model’s comprehension abilities. Additionally, RoBERTa uses a larger pretraining dataset and a bigger batch size, resulting in superior performance. It is reasonable to expect that replacing BERT with RoBERTa could lead to even better outcomes.

#### BiLSTM Model

The BiLSTM model is a deep learning architecture designed for processing sequential data, achieved by integrating 2 independent BiLSTM networks. Specifically, the BiLSTM model comprises 2 LSTM modules: one reads the sequence from left to right, and the other reads from right to left. Numerous studies have used bidirectional recurrent neural networks to extract local features, integrating them into global information after obtaining the latter using BERT [19,20]. A vector of length T, represented as $x_{1}$, $x_{2}$, …, $x_{t}$, serves as the input to the LSTM units, generating an output sequence of vectors $h_{1}$, $h_{2}$, …, $h_{t}$, all of equal length, through the application of nonlinear transformations learned during the training phase. Each $h_{t}$ is referred to as the activation of the LSTM at token t. The computational process of neurons in the LSTM is illustrated by Equations 1-4.


(1) $$i_{t}=\sigma \left(W_{xi}x_{t}+W_{hi}h_{t-1}+W_{ci}c_{t-1}+b_{i}\right)$$

(2) $$c_{t}=\left(1-i_{t}\right)⊙c_{t-1}+i_{t}⊙tanh(W_{xc}x_{t}+W_{hc}h_{t-1}+b_{c})$$

(3) $$o_{t}=\sigma (W_{xo}x_{t}+W_{ho}h_{t-1}+W_{co}c_{t}+b_{o})$$

(4) $$h_{t}=o_{t}⊙tanh(c_{t})$$

In the equations above, W and b are trainable parameters, σ represents the element-wise sigmoid function, and ⊙ is the element-wise product.

#### CRF Model

The CRF model is a machine learning model utilized for processing sequence data, especially in natural language processing. It typically takes a sequence of text as input and generates a corresponding sequence of hidden states as output. In the sequence labeling step of our research, there exists a dependency relationship between adjacent labels. For instance, an inside tag “I” must follow a beginning tag “B.” We incorporate a CRF layer following the BERT or BiLSTM layer to compute the optimal sequence combination. This layer considers the dependency relationships between adjacent labels, ensuring that an inside tag “I” follows a beginning tag “B” while maintaining a consistent type [21]. CRF assumes that a Markov random field has 2 sets of variables, where the X set usually represents a given value, denoting the input sequence, and Y represents the output under the given X condition as the corresponding output label. The graph of a CRF satisfies the following properties.

When we are under the global condition of X, meaning that the value of a random variable in X is fixed or given, Y follows the Markov property:


(5) $$P\left(\frac{Y_{u}}{X},Y_{v},u\neq v\right)=P\left(\frac{Y_{u}}{X},Y_{x},Y_{u}∼Y_{x}\right)$$

where $Y_{u}\sim Y_{x}$ indicates that $Y_{u}$ and $Y_{x}$ are neighbors in the graph.

### Integration Architecture

**Figure 3.**
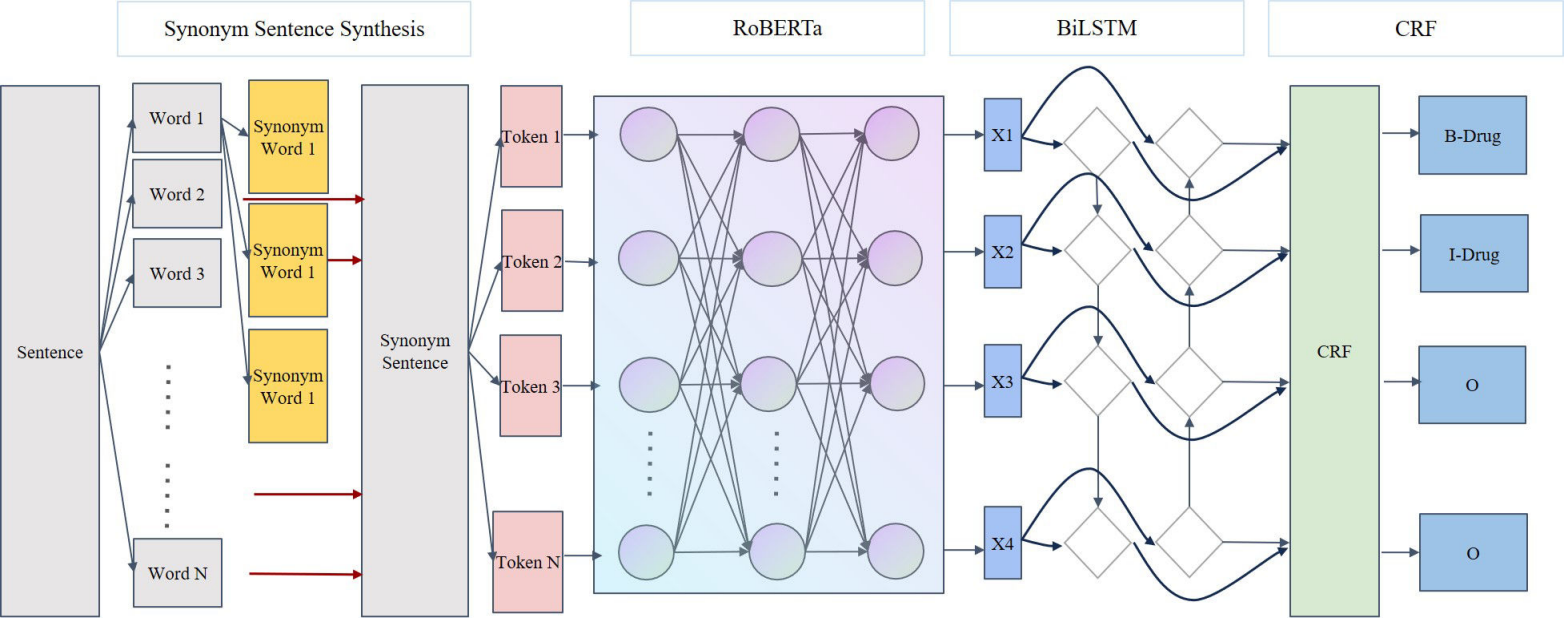
SSSS + RoBERTa + BiLSTM + CRF model structure diagram. CRF: conditional random field; BiLSTM: Bidirectional Long Short-Term Memory; RoBERTa: Robustly Optimized Bidirectional Encoder Representations from Transformers Pretraining Approach; SSSS: Segmentation Synonym Sentence Synthesis.

To evaluate the effectiveness of the SSSS algorithm compared to the original dataset, this study integrated and utilized 4 separate models (ie, BERT + CRF, BERT + BiLSTM + CRF, RoBERTa + CRF, and RoBERTa + BiLSTM + CRF). These models have similar structures but were trained using different datasets, masking representations, and training steps during the pretraining phase. The BERT + CRF and BERT + BiLSTM + CRF models have already been proven effective in numerous NER experiments [20,22,23], hence they were chosen as comparative baselines for this experiment. The impact of the downstream training set on the experimental results is significant, but the choice of pretraining dataset for the pretrained models also plays a crucial role. To validate this, the study introduced the Chinese BERT model RoBERTa, which uses more Chinese training data for model training. Finally, our model structures were divided into 2 categories, those including BiLSTM and those not including BiLSTM, as shown in Figures3  4, respectively. An ablation study was also conducted on the RoBERTa + CRF and RoBERTa + BiLSTM + CRF models.

**Figure 4.**
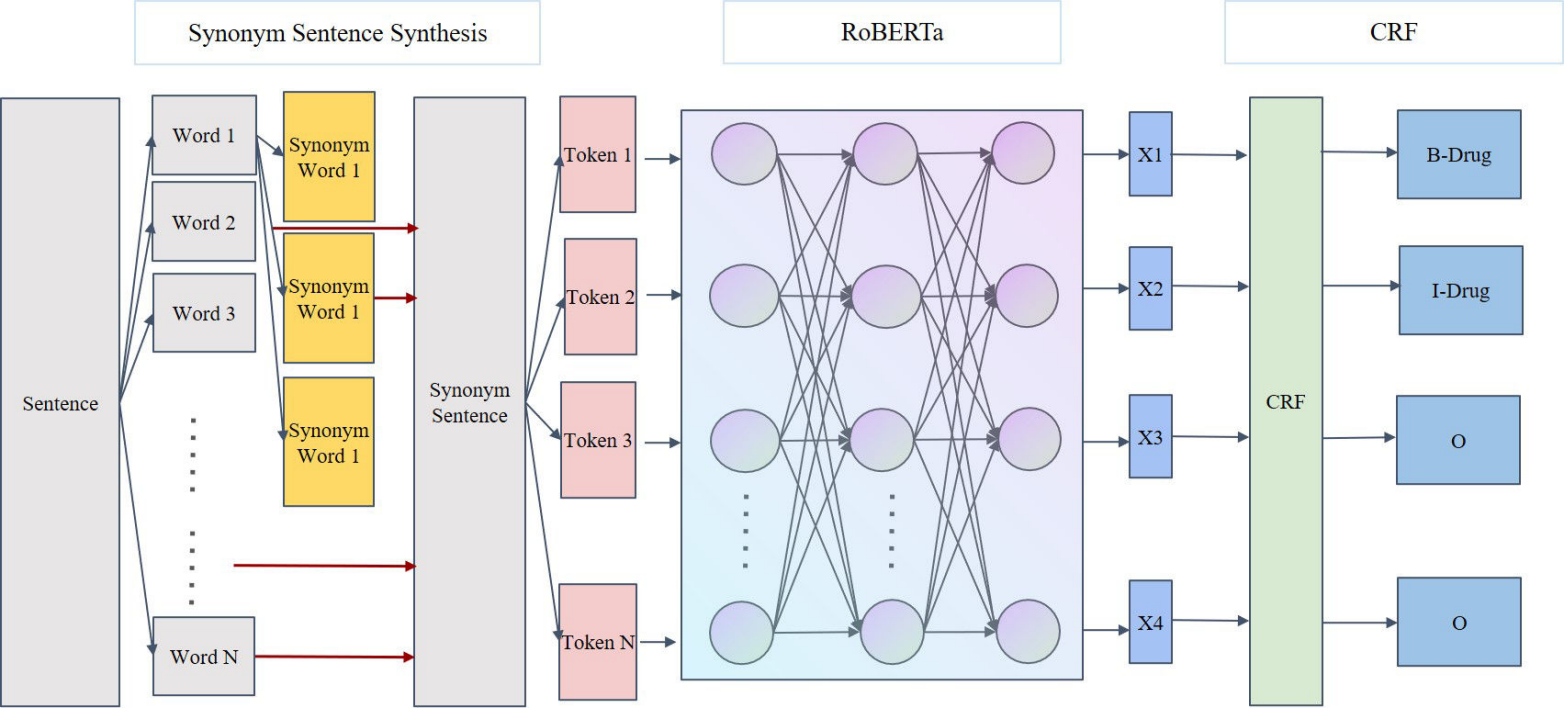
SSSS + RoBERTa + CRF model structure diagram. CRF: conditional random field; RoBERTa: Robustly Optimized Bidirectional Encoder Representations from Transformers Pretraining Approach; SSSS: Segmentation Synonym Sentence Synthesis.

### Parameter Setting

In this study, beginning, inside, outside tags are utilized to denote entities. Each clinical record may consist of several sentences and treating the record as a whole could result in excessively long samples. Therefore, we separate each record with a Chinese period. All models in this experiment were trained on a 3080 Ti GPU. Common parameter settings for all models were standardized to ensure fairness, utilizing the parameters shown in Table 2.

**Table 2.** Model parameter settings.

Parameters	Value
Learning rate of BERT/RoBERTa^a^	2×10^−5^
Learning rate of BiLSTM^b^	2×10^−5^
Learning rate of CRF^c^	2×10^−3^
Max length	256
Batch size	32
Epoch	50

a BERT/RoBERTa: Bidirectional Encoder Representations from Transformers/Robustly Optimized Bidirectional Encoder Representations from Transformers Pretraining Approach

b BiLSTM: Bidirectional Encoder Representations from Transformers.

c CRF: conditional random field.

### Ethical Considerations

The CCKS-2017 and CCKS-2019 databases used in this study are publicly available and no ethical review was required.

## Results

### Datasets

This study utilized 2 datasets from the CCKS-2017 CNER and CCKS-2019 CNER tasks, each consisting of training and testing sets. The training sets were used for model training, while the testing sets were used for model evaluation. All data were derived from progress notes and examination results in inpatient EMRs released by the CCKS challenge tasks. CCKS-2017 includes annotations for 5 entity types: symptoms, tests, diagnoses, treatments, and anatomical locations. CCKS-2019 encompasses annotations for 6 entity types: anatomical locations, surgeries, diseases, diagnoses, imaging examinations, medications, and laboratory tests. CCKS-2017 comprises 1559 training instances, while CCKS-2019 comprises 1379 training instances. The original datasets used a JSON structure to annotate the beginning and end of entities, which were then transformed into the beginning, inside, outside annotation scheme for ease of training and testing. The types and quantities of entities in the training datasets are shown in Tables3  4.

**Table 3.** Entity distribution in the China Conference on Knowledge Graph and Semantic Computing 2017 dataset.

Type	Quantity
Body	9114
Symptom	8236
Exam	11,163
Disease	1462
Treatment	3260

**Table 4.** Entity distribution in the China Conference on Knowledge Graph and Semantic Computing 2019 dataset.

Type	Quantity
Laboratory	1796
Image	1324
Operation	1194
Disease	5540
Drug	2316
Anatomy	11,521

### Evaluation Metrics

Evaluation metrics are defined by the alignment of true values and predicted results, ensuring consistency in both starting and ending positions as well as correct identification of entity types. In our experiments, we utilized precision, recall, and *F*_1_-scores to evaluate the recognition performance of the models; evaluations of all metrics were conducted at the entity level. To validate the feasibility of the SSSS algorithm, we selected dual baselines (BERT + CRF and BERT + BiLSTM + CRF) and dual datasets (CCKS-2017 and CCKS-2019), applying them simultaneously to different datasets and models to achieve cross-validation.

After applying the SSSS algorithm [24], the CCKS-2017 dataset expanded from its original size of 1559 documents to 26,768 entries, representing an expansion of approximately 17 times. Similarly, the CCKS-2019 dataset increased from its original 1379 entries to 28,933 entries, marking an expansion of approximately 20 times. The extent of entity expansion is illustrated in Tables5  6 below.

**Table 5.** Segmentation Synonym Sentence Synthesis algorithm extended effect on the China Conference on Knowledge Graph and Semantic Computing 2017 test set.

	Preexpansion	Postexpansion
Body	9114	318,220
Symptom	8236	275,457
Exam	11,163	389,045
Disease	1462	39,599
Treatment	1462	59,852

**Table 6.** Segmentation Synonym Sentence Synthesis algorithm extended effect on the China Conference on Knowledge Graph and Semantic Computing 2019 test set.

	Preexpansion	Postexpansion
Laboratory	1796	20,270
Image	1324	17,396
Operation	1194	18,662
Disease	5540	77,207
Drug	2316	24,365
Anatomy	11,521	143,332

### Experiment Results

To demonstrate the effectiveness of the algorithm, we constructed four models: (1) SSSS + BERT + CRF, (2) SSSS + BERT + BiLSTM + CRF, (3) SSSS + RoBERTa + CRF, and (4) SSSS + RoBERTa + BiLSTM + CRF. These were compared with BERT + CRF (baseline 1) and BERT + BiLSTM + CRF (baseline 2). To investigate the impact of SSSS on RoBERTa, we also performed an ablation study on the RoBERTa + CRF and RoBERTa + BiLSTM + CRF models. The results for CCKS-2017 and CCKS-2019 are presented in Tables7  8. Specifically, incorporating SSSS into the BERT + CRF and BERT + BiLSTM + CRF models resulted in *F*_1_ measure increases of 1.97% (compared with baseline 1) and 1.77% (compared with baseline 1), respectively, for CCKS-2017. Switching from BERT to RoBERTa, which includes more Chinese data in its pretraining, led to even more significant improvements. The *F*_1_-score of SSSS + RoBERTa + CRF improved by 2.51% (compared with baseline 1) and 2.36% (compared with RoBERTa + CRF), and SSSS + RoBERTa + BiLSTM + CRF improved by 2.37% (compared with baseline 2) and by 1.66% (compared with RoBERTa + BiLSTM + CRF). For CCKS-2019, similar enhancements were observed, with increases of 2.06% (compared with baseline 1) and 2.29% (compared with baseline 2) for SSSS + BERT + CRF and SSSS + BERT + BiLSTM + CRF; 2.62% (compared with baseline 1) and 2.24% (compared with RoBERTa + CRF) for SSSS + RoBERTa + CRF; and 2.44% (compared with baseline 2) and 2.12% (compared with RoBERTa + BiLSTM + CRF) for SSSS + RoBERTa + BiLSTM + CRF.

**Table 7.** Results of various methods on the China Conference on Knowledge Graph and Semantic Computing 2017 test set.

Method	Precision, %	Recall, %	*F*_1_-score, %
BERT^a^ + CRF^b^ (baseline1)	87.61	90.00	88.79
BERT + BiLSTM^c^ + CRF (baseline 2)	89.27	88.69	88.98
RoBERTa^d^ + CRF	87.52	90.40	88.94
RoBERTa + BiLSTM + CRF	89.96	89.43	89.69
SSSS^e^ + BERT + CRF	91.20	90.33	90.76
SSSS + BERT + BiLSTM + CRF	90.70	90.80	90.75
SSSS + RoBERTa + CRF	91.31	91.29	91.30
SSSS + RoBERTa + BiLSTM + CRF	91.22	91.48	91.35

a BERT: Bidirectional Encoder Representations from Transformers.

b CRF: conditional random field.

c BiLSTM: Bidirectional Long Short-Term Memory.

d RoBERTa: Robustly Optimized Bidirectional Encoder Representations from Transformers Pretraining Approach.

e SSSS: Segmentation Synonym Sentence Synthesis.

**Table 8.** Results of various methods on the China Conference on Knowledge Graph and Semantic Computing 2019 test set.

Method	Precision, %	Recall, %	*F*_1_-score, %
BERT^a^ + CRF^b^ (baseline 1)	78.43	82.88	80.59
BERT + BiLSTM^c^ + CRF (baseline 2)	78.14	83.17	80.57
RoBERTa^d^ + CRF	78.10	84.06	80.97
RoBERTa + BiLSTM + CRF	79.82	82.00	80.89
SSSS^e^ + BERT + CRF	81.08	84.28	82.65
SSSS + BERT + BiLSTM + CRF	81.22	84.57	82.86
SSSS + RoBERTa + CRF	81.10	85.46	83.21
SSSS + RoBERTa + BiLSTM + CRF	81.51	84.57	83.01

a BERT: Bidirectional Encoder Representations from Transformers.

b CRF: conditional random field.

c BiLSTM: Bidirectional Long Short-Term Memory.

d RoBERTa: Robustly Optimized Bidirectional Encoder Representations from Transformers Pretraining Approach.

e SSSS: Segmentation Synonym Sentence Synthesis.

**Figure 5.**
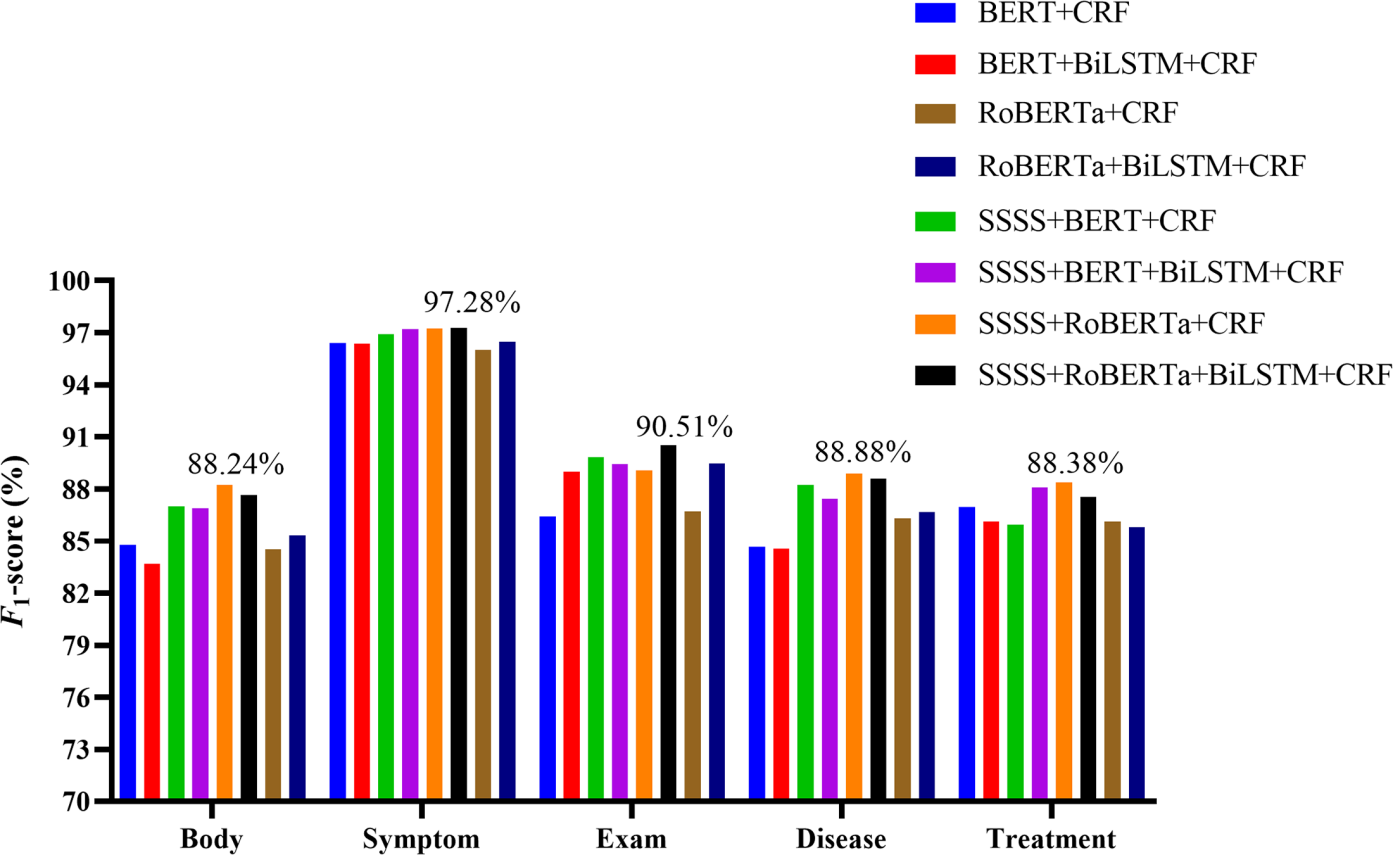
Results of different models on various entity types within the CCKS-2017 test set. BERT: Bidirectional Encoder Representations from Transformers; BiLSTM: Bidirectional Long Short-Term Memory; CCKS: China Conference on Knowledge Graph and Semantic Computing; CRF: conditional random fields; RoBERTa: Robustly Optimized Bidirectional Encoder Representations from Transformers Pretraining Approach; SSSS: Segmentation Synonym Sentence Synthesis.

Further analysis across different entity types in both datasets confirmed the comprehensive performance of our models. The experiment results are shown in Figures5  6 and Tables9  10. In CCKS-2017, all entity types showed improvements in *F*_1_-scores after applying the SSSS algorithm. Notably, the body entity type reached an *F*_1_ score of 88.24% with SSSS + RoBERTa + CRF, marking a 3.45% increase (compared with baseline 1) and 3.71% increase (compared with RoBERTa + CRF). The symptom entity type achieved its highest *F*_1_-score at 97.28% with SSSS + RoBERTa + BiLSTM + CRF, improving by 0.92% (compared with baseline 2) and 0.81% (compared with RoBERTa + BiLSTM + CRF). SSSS + RoBERTa + BiLSTM + CRF also led in the exam entity type with an *F*_1_-score of 90.51%, representing a 1.5% increase compared with baseline 2 and a 1.02% increase compared with RoBERTa + BiLSTM + CRF. The disease entity type saw their highest *F*_1_-score of 88.88% with SSSS + RoBERTa + CRF, increasing by 4.22% (compared with baseline 1) and 2.56% (compared with RoBERTa + CRF). The treatment entity achieved the highest *F*_1_-score of 88.38% using SSSS + RoBERTa + CRF, marking an increase of 1.41% (compared with baseline 1) and 2.23% (compared with RoBERTa + CRF). The CCKS-2019 results echoed this pattern of improvement across all entity types. The laboratory, image, operation, disease, drug, and anatomy entity types all saw their best performances with our models, showcasing the effectiveness of the SSSS algorithm in enhancing model accuracy and robustness.

**Figure 6.**
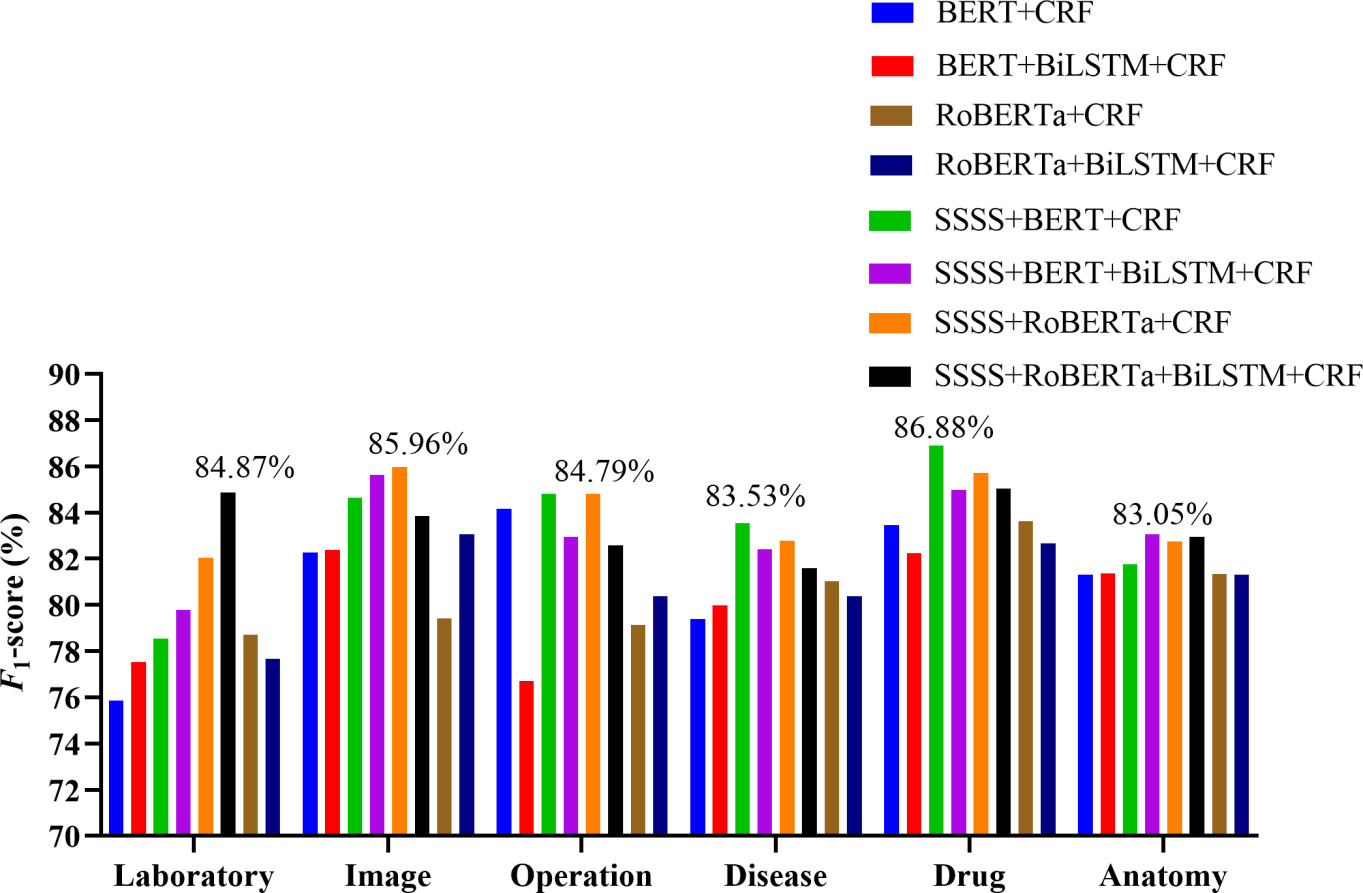
Results of different models on various entity types within the CCKS-2019 test set. BERT: Bidirectional Encoder Representations from Transformers; BiLSTM: Bidirectional Long Short-Term Memory; CCKS: China Conference on Knowledge Graph and Semantic Computing; CRF: conditional random fields; RoBERTa: Robustly Optimized Bidirectional Encoder Representations from Transformers Pretraining Approach; SSSS: Segmentation Synonym Sentence Synthesis.

**Table 9.** Results of entity type on the China Conference on Knowledge Graph and Semantic Computing 2017 test set.

Model	Body	Symptom	Exam	Disease	Treatment
BERT^a^ + CRF^b^ (baseline 1)	84.79	96.39	86.44	84.66	86.97
BERT + BiLSTM^c^ + CRF (baseline 2)	83.68	96.36	89.01	84.56	86.14
RoBERTa^d^ + CRF	84.53	96.02	86.73	86.32	86,15
RoBERTa + BiLSTM + CRF	85.34	96.47	89.49	86.68	85.82
SSSS^e^ + BERT + CRF	87.01	96.91	89.83	88.25	85.96
SSSS + BERT + BiLSTM + CRF	86.91	97.21	89.42	87.45	88.10
SSSS + RoBERTa + CRF	88.24	97.24	89.06	88.88	88.38
SSSS + RoBERTa + BiLSTM + CRF	87.65	97.28	90.51	88.61	87.55

a BERT: Bidirectional Encoder Representations from Transformers.

b CRF: conditional random field.

c BiLSTM: Bidirectional Long Short-Term Memory.

d RoBERTa: Robustly Optimized Bidirectional Encoder Representations from Transformers Pretraining Approach.

e SSSS: Segmentation Synonym Sentence Synthesis.

**Table 10.** Results of entity type on the China Conference on Knowledge Graph and Semantic Computing 2019 test set.

Model	Laboratory	Image	Operation	Disease	Drug	Anatomy
BERT^a^ + CRF^b^ (baseline 1)	75.85	82.25	84.16	79.39	83.44	81.30
BERT + BiLSTM^c^ + CRF (baseline 2)	77.54	82.39	76.71	79.97	82.25	81.36
RoBERTa^d^ + CRF	78.70	79.43	79.13	81.02	83.61	81.33
RoBERTa + BiLSTM + CRF	77.65	83.05	80.37	80.38	82.66	81.31
SSSS^e^ + BERT + CRF	78.55	84.64	84.79	83.53	86.88	81.77
SSSS + BERT + BiLSTM + CRF	79.78	85.63	82.95	82.40	84.98	83.05
SSSS + RoBERTa + CRF	82.05	85.96	84.79	82.79	85.71	82.74
SSSS + RoBERTa + BiLSTM + CRF	84.87	83.85	82.57	81.60	85.03	82.95

a BERT: Bidirectional Encoder Representations from Transformers.

b CRF: conditional random field.

c BiLSTM: Bidirectional Long Short-Term Memory.

d RoBERTa: Robustly Optimized Bidirectional Encoder Representations from Transformers Pretraining Approach.

e SSSS: Segmentation Synonym Sentence Synthesis.

To validate the performance of our model in handling unknown and low-frequency entities, we conducted experiments comparing our models (SSSS + RoBERTa + CRF and SSSS + RoBERTa + BiLSTM + CRF) with BERT + CRF and BERT + BiLSTM + CRF in terms of precision. Entities were categorized based on their occurrence frequency in the training set, as follows:

1. Unknown entities: occurrence frequency of 0 in the training set.
2. Low-frequency entities: occurrence frequency <5 times in the training set.
3. High-frequency entities: occurrence frequency ≥5 times in the training set.

The comparison results are shown in Tables11  12. From the tables, it can be observed that in the CCKS-2017 task, compared to the baseline models, our models SSSS + RoBERTa + CRF and SSSS + RoBERTa + BiLSTM + CRF improved *F*_1_-scores for unknown entities by 6.04% (compared with baseline 1) and 5.54% (compared with baseline 2), respectively. For low-frequency entities, the improvements were 7.74% (compared with baseline 1) and 6.39% (compared with baseline 2), respectively. As for high-frequency entities, improvements of 1.96% (compared with baseline 1) and 1.85% (compared with baseline 2) were achieved, respectively. Similar results were obtained in the CCKS-2019 task. Compared with the baseline models, SSSS + RoBERTa + CRF and SSSS + RoBERTa + BiLSTM + CRF achieved improvements of 4.21% (compared with baseline 1) and 2.29% (compared with baseline 2) for unknown entities, respectively, for . For low-frequency entities, improvements of 2.35% (compared with baseline 1) and 6.31% (compared with baseline 2) were achieved, while for high-frequency entities, improvements of 1.09% (compared with baseline 1) and 0.95% (compared with baseline 2) were observed. These results demonstrate significant enhancements in handling unknown and low-frequency entities after expanding the training dataset, with more noticeable improvements observed for low-frequency entities compared to unknown entities.

**Table 11.** The *F*_1_-scores for each method on the China Conference on Knowledge Graph and Semantic Computing 2017 test set.

Model	Unknown entities	Low-frequency entities	High-frequency entities
BERT^a^ + CRF^b^ (baseline 1)	40.95	53.43	91.96
BERT + BiLSTM^c^ + CRF (baseline 2)	42.59	55.98	92.09
SSSS^d^ + RoBERTa^e^ + CRF	46.99	61.17	93.92
SSSS + RoBERTa + BiLSTM + CRF	48.13	62.37	93.94

a BERT: Bidirectional Encoder Representations from Transformers.

b CRF: conditional random field.

c BiLSTM: Bidirectional Long Short-Term Memory.

d SSSS: Segmentation Synonym Sentence Synthesis.

e RoBERTa: Robustly Optimized Bidirectional Encoder Representations from Transformers Pretraining Approach.

**Table 12.** The *F*_1_-scores for each method on the China Conference on Knowledge Graph and Semantic Computing 2019 test set.

Model	Unknown entities	Low-frequency entities	High-frequency entities
BERT^a^ + CRF^b^ (baseline 1)	47.84	63.90	83.65
BERT + BiLSTM^c^ + CRF (baseline 2)	45.58	63.59	84.01
SSSS^d^ + RoBERTa^e^ + CRF	52.05	66.25	84.74
SSSS + RoBERTa + BiLSTM + CRF	47.87	68.68	84.96

a BERT: Bidirectional Encoder Representations from Transformers.

b CRF: conditional random field..

c BiLSTM: Bidirectional Long Short-Term Memory.

d SSSS: Segmentation Synonym Sentence Synthesis.

e RoBERTa:Robustly Optimized Bidirectional Encoder Representations from Transformers Pretraining Approach.

To demonstrate the superiority of our model, we compared it with existing state-of-the-art models. Table 13 presents the experimental results of different models on the CCKS-2017 and CCKS-2019 datasets. Our model shows a clear advantage.

**Table 13.** Comparison of results with existing models on the China Conference on Knowledge Graph and Semantic Computing 2017 and 2019 datasets.

Model	2017 dataset			2019 dataset		
	Precision, %	Recall, %	*F*_1_-score, %	Precision, %	Recall, %	*F*_1_-score, %
AT^a^-Lattice LSTM^b^-CRF^c^ [25]	88.98	90.28	89.64	—^d^	—	—
BiLSTM^e^-CRF + Gazetteer + Spatial Attention [26]	85.39	87.62	86.49	—	—	—
BiLSTM-Att^f^-CRF + POS^g^ + Dic^h^ [27]	90.41	90.49	90.48	—	—	—
MCBERT^i^-GCN^j^-CRF [28]	—	—	—	83.87	82.26	83.06
SSSS^k^ + RoBERTa^l^ + CRF	91.31	91.29	91.30	81.10	85.46	83.21
SSSS + RoBERTa + BiLSTM + CRF	91.22	91.48	91.35	81.51	84.57	83.01

a AT: adversarial training.

b LSTM: Long Short-Term Memory.

c CRF: conditional random field.

d Not applicable.

e BiLSTM: Bidirectional Long Short-Term Memory.

f Att: attention.

g POS: part-of-speech.

h Dic: dictionary.

i MCBERT: Medical Chinese Bidirectional Encoder Representations from Transformers.

j GCN: graph neural network.

k SSSS: Segmentation Synonym Sentence Synthesis.

l RoBERTa: Robustly Optimized Bidirectional Encoder Representations from Transformers Pretraining Approach.

## Discussion

### Principal Results

We proposed the SSSS algorithm based on neighboring vocabulary to effectively expand the training dataset without introducing additional specialized domain dictionaries, thereby enhancing the model’s performance in CNER tasks. The algorithm utilized the Jieba library to tokenize the original entities, then used a natural language vocabulary trained based on Word2Vec and calculated neighboring vocabulary through the Synonyms library to generate more forms of entity expressions, which are integrated into the training set. This approach allowed the model to encounter more diverse forms of entities during training, thereby improving its generalization ability and capability to recognize diverse entities.

In terms of model structure, this study adopted BERT as the underlying model, combined with the CRF model for sequence labeling tasks, and introduced the BiLSTM model for extracting local features. Experimental results demonstrated that these models achieved significant performance improvement in handling CNER tasks after introducing the SSSS algorithm. The algorithm substantially augmented the dataset, leading to notable enhancements in identifying previously unknown entities and low-frequency entities. Particularly, the improvement in low-frequency entities was substantial, as the generation of expanded entities depends on the decomposition and recombination of existing entities. By splitting and expanding low-frequency entities, their frequencies can be increased, effectively enhancing the model’s recognition capabilities for these entities. For example, in the EMR text “依据头颅 CT：多发脑梗死，故多发脑梗死诊断明确 (Based on cranial CT: multiple cerebral infarctions, hence the diagnosis of multiple cerebral infarctions is clear),” the disease entity “多发脑梗死 (multiple cerebral infarctions)” and the treatment entity “单硝酸异山梨酯扩冠 (isosorbide mononitrate vasodilation)” in the phrase “单硝酸异山梨酯扩冠改善心肌缺血 (isosorbide mononitrate vasodilation to improve myocardial ischemia)” appeared only once in the original dataset and they were not recognized by the baseline model. However, after SSSS expansion, these entities were successfully identified. For high-frequency entities, such as the cure entities “阿司匹林 (Aspirin)” and “头孢哌酮钠舒巴坦钠 (Cefoperazone Sodium and Sulbactam Sodium)” and the disease entity “冠心病 (coronary heart disease),” expansion further increased their occurrence frequency in the training set, improving coverage. However, for previously unknown entities, although some new entities could be generated through the decomposition and expansion of high-frequency and low-frequency entities, their improvement was less than that of low-frequency entities. For example, the body entity “右侧胸腔 (right pleural cavity)” did not exist in the original dataset but was successfully identified through expansion from entities like “胸腔 (pleural cavity)” and “左侧胸腔 (left pleural cavity).” However, drug entities such as “地高辛 (digoxin)” and “格列本脲 (glibenclamide),” which were also absent in the original dataset, remained unrecognized even after expansion. This is because it is difficult to create entities that are entirely absent from the original training set but that exist in the medical domain; these entities are far from any entity in the original training set based on the edit distance algorithm. Subsequently, replacing BERT with RoBERTa further improved performance, attributed to RoBERTa’s increased use of pretraining data, leading to increased data volume and iteration rounds, thus validating the effectiveness and superiority of the proposed model.

This study adopted a multibaseline and multidataset cross-experimental method, achieving significant improvements in 2 model structures (BERT + CRF and BERT + BiLSTM + CRF) and 2 datasets (CCKS-2017 and CCKS-2019), demonstrating that the method of expanding the dataset by replacing neighboring vocabulary expressions with new words can effectively improve the accuracy and recall of the model on vocabulary in different models.

### Limitations and Future Work

The increase in training time due to the expansion of vocabulary expressions varies. Moreover, it can be observed that in the CCKS-2019 task, the use of the expanded dataset for anatomical entities was improved but still did not reach the average level. This may be because anatomical entities often appear mixed in surgical or disease and diagnosis entities. Additionally, since the algorithm did not introduce additional domain dictionaries, there are still shortcomings in the expansion method for discovering new unknown entities. Due to the extensive expansion of domain-specific vocabulary, it may be difficult to ensure that the restructured sentences fully retain the original meaning. With the rapid development of medical information, EMR text data are becoming increasingly extensive and complex, resulting in higher requirements for the performance and efficiency of models. In future research, further combining small-scale domain dictionaries to enhance the coverage of unknown entities—or using techniques such as random word replacement with MacBERT or Chinese word embeddings with BERT-wwm—while addressing issues like nested anatomical entities and Chinese word segmentation ambiguities remains a direction that requires continued exploration and investigation.

### Conclusion

This study introduces an adaptive dataset optimization algorithm named SSSS, which is based on the utilization of nearby vocabulary expressions. The algorithm was extensively validated using the CCKS-2017 and CCKS-2019 datasets. We leveraged existing public knowledge, eliminating the need for manual expansion of specialized domain dictionaries. By segmenting the existing vocabulary and replacing it with new synonyms from the large natural language database word2vec, we achieved the recombination of the datasets’ nearby expanded expressions. Experimental results demonstrated that our algorithm successfully expanded the documents of CCKS-2017 and CCKS-2019 by approximately 17 times and 20 times, effectively addressing challenges such as data acquisition, annotation difficulties, and insufficient model generalization performance.

In terms of performance evaluation, when compared to the basic BERT + CRF and BERT + BiLSTM + CRF models, our model improved *F*_1_-scores by 2.51% and 2.37% in the CCKS-2017 task, and achieved an increase of 2.62% and 2.44% in *F*_1_-scores in the CCKS-2019 task. Furthermore, through the expansion of nearby vocabulary, our model outperformed BERT + CRF and BERT + BiLSTM + CRF in handling unknown entities and low-frequency entities. This provides a novel approach for addressing challenges in CNER tasks, such as the unstructured nature of clinical text, poor contextual association, and difficulties in annotation.

## Abbreviations

BERT: Bidirectional Encoder Representations from Transformers
BiLSTM: Bidirectional Long Short-Term Memory
CCKS: China Conference on Knowledge Graph and Semantic Computing
CNER: clinical named entity recognition
CRF: conditional random field
Dic-Att-BiLSTM-CRF: dictionary-attention-Bidirectional Long Short-Term Memory-conditional random field
EMR: electronic medical record
LSTM: Long Short-Term Memory
NER: named entity recognition
RoBERTa: Robustly Optimized Bidirectional Encoder Representations from Transformers Pretraining Approach
SSSS: Segmentation Synonym Sentence Synthesis
